# Circular RNAs Regulate Vascular Remodelling in Pulmonary Hypertension

**DOI:** 10.1155/2022/4433627

**Published:** 2022-11-03

**Authors:** Shuang-Lan Xu, Jie Liu, Shuang-Yan Xu, Ze-Qin Fan, Yi-Shu Deng, Li Wei, Xi-Qian Xing, Jiao Yang

**Affiliations:** ^1^Department of Respiratory Medicine, The Affiliated Hospital of Yunnan University, The Second People's Hospital of Yunnan Province, Kunming, 650021 Yunnan, China; ^2^Department of Dermatology and Venereology, The Second Affiliated Hospital of Kunming Medical University, Kunming, Yunnan, China; ^3^Department of Dermatology, The People's Hospital of Yuxi City, The Sixth Affiliated Hospital of Kunming Medical University, Yuxi 653100, Yunnan, China; ^4^First Department of Respiratory Medicine, The First Affiliated Hospital of Kunming Medical University, Kunming, 650032 Yunnan, China

## Abstract

Circular RNAs (circRNAs) are a newly identified type of noncoding RNA molecule with a unique closed-loop structure. circRNAs are widely expressed in different tissues and developmental stages of many species, participating in many important pathophysiological processes and playing an important role in the occurrence and development of diseases. This article reviews the discovery, characteristics, formation, and biological function of circRNAs. The relationship between circRNAs and vascular remodelling, as well as the current status of research and potential application value in pulmonary hypertension (PH), is discussed to promote a better understanding of the role of circRNAs in PH. circRNAs are closely related to the remodelling of vascular endothelial cells and vascular smooth muscle cells. circRNAs have potential application prospects for in-depth research on the possible pathogenesis and mechanism of PH. Future research on the role of circRNAs in the pathogenesis and mechanism of PH will provide new insights and promote screening, diagnosis, prevention, and treatment of this disease.

## 1. Introduction

Pulmonary hypertension (PH) refers to a haemodynamic state of abnormally elevated pulmonary artery pressure. The pathological characteristics of PH primarily involve pulmonary vascular remodelling, which is accompanied by varying degrees of wall thickening, lumen stenosis, and increased vascular resistance, eventually leading to right heart failure and death [1]. The occurrence and development of PH is a complex process involving multiple factors and molecules and is affected by genetic susceptibility and various endogenous and exogenous stimuli [2]. PH is characterized by an inflammatory response and pulmonary arterial endothelial cell (PAEC) dysfunction, and pulmonary artery smooth muscle cell (PASMC) proliferation, migration, and apoptosis caused by pulmonary vascular structure reconstruction are key aspects in PH development [3]. Important advances in research on the pathogenesis and prevention of PH have been made in the past decade, and most strategies mainly dilate pulmonary vessels and cannot completely reverse pulmonary vessel remodelling. However, the mechanism of the occurrence and development of PH is complex, and PH-associated morbidity and mortality are still increasing every year. This increase has resulted in a heavy burden on society and families and has become a serious threat to the health of individuals with major underlying diseases [4].

Noncoding RNAs (ncRNAs) were originally considered to be “noise” from transcription that lacked any important biological effects. However, increasing evidence shows that ncRNAs are associated with complex human diseases [5–7]. Circular RNAs (circRNAs), newly identified ncRNA molecules, are involved in the occurrence and development of human diseases, and they can be used as potential biomarkers for diagnosis and prognosis [5, 8, 9]. Notably, circRNAs have a crucial regulatory role in respiratory diseases [10]. Recently, researchers systematically collected and reviewed reports describing the functions of ncRNAs (miRNAs, lncRNAs, and circRNAs) in PH [10–13]. However, to the best of our knowledge, there is no systematic review of circRNAs that regulate vascular remodelling of PASMCs and PAECs in PH. Therefore, in this review, the research status and potential application value of circRNAs in PH are discussed. The abnormal expression of circRNAs is involved in the pathogenic mechanism of vascular remodelling in PH and provides novel biomarkers for diagnosis and treatment.

## 2. Overview of circRNAs

### 2.1. Discovery and Characteristics of circRNAs

circRNAs were first discovered in plant viruses by electron microscopy in the early 1970s [14]. As circRNAs do not encode proteins, they initially did not attract the attention of researchers and were considered “junk sequences” resulting from transcriptional “noise” [15]. However, with the rapid development of RNA high-throughput sequencing technology and bioinformatics, circRNAs have been shown to be widespread in different species, where they are highly conserved, tissue-specific, and timing-specific molecules that have important biological functions [16]. circRNAs are not linear structures but rather exhibit a closed-loop structure without a 5′ end cap or a 3′ end tail, exhibiting resistance to exonucleases and a high degree of stability [17].

### 2.2. Formation of circRNAs

circRNAs primarily exist in the cytoplasm and are low-abundance RNA molecules formed by the incorrect splicing of exon transcripts. Unlike the typical splicing of mRNA, circRNAs are formed by reverse splicing. Most circRNAs are generated by exon circularization, with some circRNAs being formed by intron circularization, but they are all transcribed from pre-mRNA sequences by RNA polymerase. Thus, circRNAs primarily comprise exon-derived circRNAs, intron-derived circRNAs, and exon–intron circRNAs [18, 19].

### 2.3. Functions of circRNAs

Previous studies have shown that the potential functions of circRNAs include acting as miRNA sponges to compete with endogenous RNAs (ceRNAs), regulating gene transcription and protein binding, and encoding proteins. These aspects are discussed in the following sections.

### 2.4. circRNAs as Competitive Endogenous RNAs

In 2013, Memczak et al. [16] and Hansen et al. [20] proposed, for the first time, that circRNAs are rich in miRNA binding sites that can be used as natural sponges for adsorbing miRNAs. This activity prevents miRNAs from interacting with mRNAs in the 3′ untranslated region, thereby indirectly regulating the downstream target gene expression of miRNAs, a mechanism that is defined as the competitive endogenous RNA (competing endogenous RNA, ceRNA) hypothesis [21]. The proposition of this hypothesis has led to changes in the understanding of the regulatory mode of miRNAs during the regulation of gene expression, from classic miRNAs-mRNAs to circRNAs-miRNAs-mRNAs, which have received increasing attention in the study of disease pathogenesis.

### 2.5. Regulation of Gene Transcription

circRNAs can participate in regulation at the transcriptional and posttranscriptional levels. Some studies have noted that intron-derived circRNAs interact with RNA polymerase II and have a cisregulatory effect on the transcription of parental encoding genes [22], while exons and introns composed of circRNAs have been shown to bind to small ribonucleoproteins in a complex that interacts with RNA polymerase II in the promoter region to enhance gene transcription [19]. These two types of RNA primarily play a role in the nucleus. In addition, circRNAs compete with other RNAs through sponge adsorption to bind RNA-binding proteins or miRNAs, thereby inhibiting their function and participating in the regulation of posttranscriptional processes [16, 23].

### 2.6. Regulation of Protein Binding

circ-Foxo3 is a circRNA encoded by the tumour suppressor gene Foxo3 that can combine with different proteins to cause different biological effects. circ-Foxo3 binds to cyclin-dependent kinase inhibitor 1 (p21) and cyclin-dependent kinase 2 (CDK2) to form a ternary complex of protein circ-Foxo3-p21-CDK2, thereby inhibiting CDK2 and cyclin A binding to cyclin E and preventing cell cycle progression [24]. circ-Foxo3 binds to the antiaging protein ID-1, the transcription factor E2F1, and the antistress proteins FAK and HIF1*α* in the cytoplasm, inhibiting the ageing and antistress effects of these proteins and leading to increased cell ageing [25].

### 2.7. Encoding Proteins

Although circRNAs are noncoding RNAs, some of them also encode proteins. As early as the 20th century, some scholars reported that a class of circRNAs containing internal ribosome entry site elements can be translated by eukaryotic ribosomes and synthesize polypeptide chains [26]. In recent years, additional studies have confirmed this function of circRNAs. circ-ZNF609 in human- and mouse-derived myoblasts contains an open reading frame, similar to a linear transcript, starting from the start codon and ending at the frame. The internal stop codon, under the action of polysomes, is translated into protein, resulting in specific control of myoblast proliferation [27].

In addition, circRNAs can compete to regulate the process of alternative splicing during reverse splicing, subsequently regulating the expression of host genes [28]. Other unknown functions of circRNAs need to be identified in the future.

## 3. circRNAs and Vascular Remodelling

**Figure 1:**
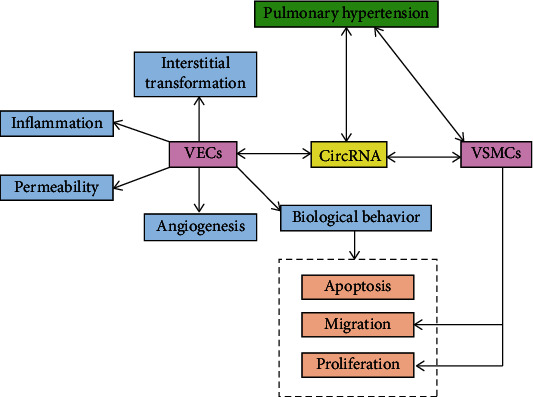
Regulatory relationship between circular RNA and vascular remodelling in pulmonary hypertension.

Pulmonary vascular remodelling is an important part of PH development, and it is essential to understand the progression and mechanism of this process. The pulmonary vascular wall has three layers: the outer layer, which contains fibroblasts; the middle layer, which consists of smooth muscle cells and one or more elastic layers; and the inner layer, which is characterized by a single layer of endothelial cells. Pulmonary vascular remodelling is the process of the thickening of the pulmonary vascular wall due to hypertrophy (cell growth) or hyperplasia (cell proliferation) of one or more cell types and the increase in extracellular matrix components. This process involves all layers of the vascular wall [29, 30], with endothelial dysfunction and abnormal smooth muscle cell proliferation being the most common.  Figure 1 shows the regulatory relationship between circRNA and vascular remodelling in PH.

### 3.1. circRNAs and Vascular Endothelial Cells

Many differentially expressed circRNAs can be detected in vascular endothelial cells (VECs) from different sources. These circRNAs regulate various functions of VECs and are important molecules for the onset of various diseases, providing a new approach to disease diagnosis and a novel treatment target.

circRNAs regulate the proliferation, migration, apoptosis, and angiogenesis of VECs. Furthermore, circRNAs can promote or inhibit the growth, migration, apoptosis, and angiogenesis of VECs derived from umbilical veins, aorta, or other blood vessels and participate in the pathogenesis of diabetes, hypertension, atherosclerosis, and other diseases [31–34]. In addition, the influence of circRNAs on the angiogenesis of VECs promotes the occurrence of diseases closely related to this function, such as corneal neovascularization and gliomas [35, 36].

circRNAs are involved in regulating the epithelial-mesenchymal transition process that involves the transformation of VECs into mesenchymal cells, where VECs lose their original phenotypic characteristics and obtain the motility and contraction characteristics of mesenchymal cells. Neuroinflammatory diseases, bladder cancer, and pulmonary fibrosis play an important role in the occurrence and development of diseases [37–39].

circRNAs promote inflammation in VECs. To date, the role of circRNAs in the inflammatory response of VECs has only been reported in atherosclerotic diseases. The mechanism by which circ-RELL1 promotes the development of atherosclerosis is achieved by a ceRNA regulating the miR-6873-3p/MyD88/NF-*κ*B axis [40], while circ-ANRIL increases the number of inflammatory factors to promote atherosclerosis [41].

In addition, circRNAs can regulate the permeability of the vascular endothelium. Li et al. showed that circ-IARS enters human microvascular venous VECs through exosomes from pancreatic cancer cells, increasing the permeability of the endothelial layer and thereby promoting the invasion and metastasis of pancreatic cancer [42].

### 3.2. circRNAs and Vascular Smooth Muscle Cells (VSMCs)

VSMCs are highly differentiated cells that are primarily present in the tunica media of arteries and arterioles and have extremely high phenotypic plasticity. Under pathophysiological conditions, these cells can transition from a differentiated/contracted state to a dedifferentiated/proliferative state, resulting in increased migration and proliferation.

At present, circRNA research is primarily focused on regulating the biological behaviours of VSMCs, such as their proliferation and migration, and their involvement in the occurrence and development of diseases. Hall et al. observed that 256 circRNAs were specifically expressed in mouse VSMCs by mining the annotated mouse circRNAs in the circBase database [43]. Bioinformatics analysis revealed that the abnormal expression of circ_Lrp6 is most closely related to the function of VSMCs. Later, experimental studies showed that silencing circ_Lrp6 can reduce the proliferation and migration of VSMCs in vitro and promote their differentiation, while inhibiting circ_Lrp6 in mice could reduce the formation of neointimal vasculature [43, 44], which is consistent with previous bioinformatics predictions. In addition, circCHFR was confirmed to be abnormally overexpressed in arterial smooth muscle cells (ASMCs). Cell function experiments showed that the interference of circCHFR can inhibit the proliferation and migration of ASMCs and is involved in the regulation of phenotypic changes in ASMCs. Therefore, circCHFR is considered to be a promoter of atherosclerosis [45], while circACTA2 can regulate the contraction of human ASMCs and the expression of *α*-smooth muscle actin (*α*-SMA) through the NRG-1-ICD/circACTA2/miR-548f-5p axis [46].

## 4. Role of circRNAs in PH

PH-associated circRNAs were initially identified by microarray analysis. In two experiments, Miao et al. extracted blood samples from individuals with chronic thromboembolic pulmonary hypertension (CTEPH) and healthy control human blood samples and detected abnormally expressed circRNAs. Subsequently, along with the bioinformatics analysis results, these researchers observed that hsa_circ_0002062, hsa_circ_0022342, and hsa_circ_0046159 can competitively target specific miRNAs and enrich them in important signalling pathways related to CTEPH. These circRNAs play an important role in the development and targeted therapy of CTEPH [47, 48]. Huang et al. found that hsa_circ_0003416 in the plasma was significantly downregulated in children with pulmonary arterial hypertension (PAH) caused by congenital heart disease (CHD), and this molecule could be used as a biomarker for the diagnosis of PAH-CHD [49]. Elevated serum circ_0068481 and a lower level of circGSAP in peripheral blood mononuclear cells can be used as new noninvasive biomarkers for the diagnosis and prognosis of idiopathic PAH [50, 51]. In terms of human data, the description of tissue-expressed circRNAs in diseased human tissues is very limited, therefore requiring large-scale studies and reproducible validation of biomarker datasets. In addition, differentially expressed circRNAs were detected in the lung tissue of mice with PH induced by hypoxia [52]. Our previous study identified dysregulated circRNAs from a hypoxic PH rat model and confirmed that circRNAs acting as ceRNAs were involved in PH development [53]. However, the regulatory mechanism of circRNAs in PH has not been clarified.

According to the literature, there are few experimental studies on the mechanism of circRNAs in PH, and the dysregulated circRNAs that regulate vascular remodelling in PH are shown in Table 1. circHIPK3, mainly originating from the second exon of the gene HIPK3, is highly expressed and has an important function in cells. Recently, the circHIPK3-miR-328-3p-STAT3 axis was shown to be involved in the pathogenesis of PAH by stimulating hPAEC proliferation, migration, and angiogenesis [54]. hsa_circ_0016070 inhibits miR-942-5p through competitive targeting, increasing the expression of the downstream cyclin D1 (CCND1) gene and promoting the proliferation and cell cycle progression of primary-cultured PASMCs to regulate vascular remodelling in PH. Thus, hsa_circ_0016070 was identified as a potential new biomarker in PH diagnosis and treatment [55]. Zhang et al. performed in vivo and in vitro experiments and showed that circ-Calm4 can be used as an miR-337-3p sponge to regulate myosin 10 (Myo10), which affected the expression of cell cycle-related proteins, promoted PASMC proliferation, and exacerbated hypoxia-induced pulmonary vascular remodelling in the PH mouse model. In contrast, silencing of circ-Calm4 can inhibit the proliferation of PASMCs and reverse pulmonary vascular remodelling in mice [56]. Another study first showed that circ-Calm4 regulated cell pyroptosis of PASMCs via the circ-Calm4/miR-124-3p/PDCD6 axis, which may potentially be useful for therapeutic strategies of PH [57]. Recently, a novel study investigated a typical circRNA, CDR1as, that regulated the pathological process of vascular calcification by targeting miR-7-5p in PH [58]. Currently, dysregulated circRNAs act as ceRNAs to regulate the biological behaviour of PASMCs and PAECs, including proliferation, cell cycle progression, migration, and apoptosis [54–57, 59–63].

Furthermore, recent studies have demonstrated different functions of circRNAs in PH. circRNAs compete with other RNAs through sponge adsorption to bind RNA-binding proteins, thereby inhibiting their function and participating in the regulation of posttranscriptional processes. Downregulated circ-Sirt1 and upregulated circ-Grm1 interacted with the RNA-binding protein and led to changes in the proliferation and migration of PASMCs [64, 65]. m6A plays important roles in various biological processes. A recent study identified a transcriptome-wide map of m6A circRNAs in hypoxia-mediated PH, and m6A circRNAs were mainly from protein-coding gene spanning single exons and influencing the circRNA–miRNA–mRNA coexpression network [66]. However, this potential mechanism remains to be explored. m6A circRNAs regulate pulmonary vascular remodelling by affecting the function of PASMCs and PAECs and contribute to the occurrence and development of PH.

To date, most research on the function of PH-associated circRNAs has been limited to studies of these molecules as biomarkers or as molecular sponges that adsorb miRNAs or bind RNA-binding proteins, exert ceRNA activity, and affect the biological behaviour of PASMCs, providing a new perspective in the study of PH pathogenesis. Interestingly, recent studies concluded that computational models could effectively predict potential ncRNA-disease associations for further experimental verification, which would help save resources [67, 68]. This study may provide a new future research direction for the identification of circRNA biomarkers related to PH.

Our understanding of the role of circRNAs in PH is still preliminary. Previous studies have shown that the abnormal expression of circRNAs could help us understand the pathogenic mechanism of PH, provide new insights, and promote the screening, diagnosis, prevention, and treatment of this disease. However, to the best of our knowledge, there is no systematic review of circRNAs that regulate vascular remodelling of PASMCs and PAECs in PH. Our review focuses on circRNAs regulating vascular remodelling in PH. However, owing to the limited sample size of the studies and lack of confirmed research in vivo, the hypothesis that circRNA plays important roles in the onset and progression of PH requires further confirmation.

## 5. Conclusions

With the rapid development of genomics and bioinformatics analyses, the role of circRNAs has been recognized. The regulatory mechanisms of circRNAs in PH are still unclear. Here, we have reviewed recent studies on circRNAs that revealed their associations with PH and suggested potential mechanisms. Identification of more circRNAs involved in PH and exploration of their functions and targets are needed in the future.

## 6. Opinions

circRNAs, as novel noncoding RNA molecules with a unique closed-loop structure and many potential regulatory functions, have attracted increasing attention in different respiratory diseases, such as lung cancer, asthma, and chronic obstructive pulmonary disease. circRNAs are characterized by high stability, conservation, and abundance in peripheral blood and other body fluids and could be effective clinical diagnostic and prognostic biomarkers for respiratory diseases. At present, studies on the role of circRNAs in PH are just beginning. With the rapid advances in high-throughput sequencing techniques and bioinformatics analysis methods, most studies have identified differentially expressed circRNAs using microarray or next-generation RNA sequencing and verified that circRNAs are likely to become emerging novel clinical biomarkers; however, the function and potential molecular mechanisms of circRNAs remain unclear.

Interestingly, in vascular diseases, such as diabetes and atherosclerosis, circRNAs are closely related to the remodelling of vascular endothelial cells and VSMCs. Pulmonary vascular remodelling is an important part of PH development, and it is essential to understand the progression and mechanism of this process. In recent years, research on the function of PH-associated circRNAs has been limited to studies of these molecules as molecular sponges that adsorb miRNA or bind RNA-binding proteins, exert ceRNA activity, and affect the biological behaviour of PASMCs. New functions and molecular mechanisms of circRNAs in PH research need to be determined in the near future, and these studies will provide new insights and promote the screening, diagnosis, and prevention of PH-related diseases.

## Figures and Tables

**Table 1:** A list of dysregulated circRNAs regulates vascular remodelling in pulmonary hypertension.

circRNAs	Regulation	Species	Targets	Cells	Model construction	Functions	Reference
circWDR37_016	Upregulated	Human	miR-138	PASMCs	Hypoxia-induced	Proliferation, cell cycle progression, migration, and apoptosis	Ref. 53
circ-Sirt1	Downregulated	Rat	TGF-*β*1/Smad3/Smad7/Sirt1	PASMCs	Hypoxia-induced	Proliferation, cell cycle progression, and migration	Ref. 59
circ_0002062	Upregulated	Human	miR-942-5P/CDK6	PASMCs	Hypoxia-induced	Proliferation, migration, and apoptosis	Ref. 54
circHIPK3	Upregulated	Human	miR-328-3p/STAT3	PAECs	PDGF treatment	Proliferation, migration, and angiogenesis	Ref. 48
circ-Grm1	Upregulated	Mouse	Grm1/Rap1/ERK	PASMCs	Hypoxia-induced	Proliferation and migration	Ref. 58
circ-Calm4	Upregulated	Mouse	miR-124-3P/PDCD6	PASMCs	Hypoxia-induced	Pyroptosis, proliferation, and apoptosis	Ref. 51
circNFXL1_009	Downregulated	Human	miR-29b-2-5P/KCNB1	PASMCs	Hypoxia-induced	Proliferation, migration, and apoptosis	Ref. 55
circ-CDR1as	Upregulated	Human	miR-7-5P/CAMK2D	PASMCs	Hypoxia-induced	Osteoblastic differentiation and calcification	Ref. 52
circ-CDR1as	Upregulated	Human	miR-7-5P/CNN3	PASMCs	Hypoxia-induced	Osteoblastic differentiation and calcification	Ref. 52
circ-Calm4	Upregulated	Mouse	miR-337-3p/Myo10	PASMCs	Hypoxia-induced	Proliferation and cell cycle progression	Ref. 50
circATP2B4	Upregulated	Human	miR-223/ATR	PASMCs	Hypoxia-induced	Proliferation, migration, and apoptosis	Ref. 56
circ_0000790	Upregulated	Mouse	miR-374c/FOXC1	PASMCs	Hypoxia-induced	Proliferation, migration, and apoptosis	Ref. 57
circ_0016070	Upregulated	Human/rat	miR-942/CCND1	PASMCs	Primary culture	Proliferation and cell cycle progression	Ref. 49

PASMCs: pulmonary arterial smooth muscle cells; PAECs: pulmonary artery endothelial cells.

## Additional Points

*Article Highlights*. (i) circRNAs participate in many important pathophysiological processes and play an important role in the occurrence and development of diseases. (ii) circRNAs are closely related to the remodelling of vascular endothelial cells and vascular smooth muscle cells. (iii) circRNAs have potential application value in pulmonary hypertension.
